# Clinical outcomes of BCG‐treated patients with high‐risk non‐muscle‐invasive bladder cancer according to pre‐ and post‐2021 European Association of Urology risk classifications

**DOI:** 10.1111/bju.70247

**Published:** 2026-03-23

**Authors:** Tian Ye, Benjamin Tura, Ben Abbotts, Maurice P. Zeegers, K. K. Cheng, Nicholas D. James, Richard T. Bryan

**Affiliations:** ^1^ Bladder Cancer Research Centre, Department of Cancer and Genomic Sciences College of Medicine and Health, University of Birmingham Birmingham UK; ^2^ Department of Urology Guizhou Provincial People's Hospital Guiyang China; ^3^ Department of Epidemiology, School of Nutrition and Translational Research in Metabolism Maastricht University Maastricht The Netherlands; ^4^ Department of Applied Health Sciences, College of Medicine and Health University of Birmingham Birmingham UK; ^5^ Institute of Cancer Research London UK

**Keywords:** Non‐muscle‐invasive bladder cancer, Bacillus Calmette‐Guérin, progression, risk stratification, European Association of Urology, BCG, clincial outcomes

**Abbreviations**
CIS: carcinoma *in situ*
EAU: European Association of Urology
HR: high risk
IQR: interquartile range
IR: intermediate risk
NMIBC: non‐muscle‐invasive bladder cancer
PFS: progression‐free survival
RC: radical cystectomy
sHR: sub‐hazard ratio
TURBT: transurethral resection of bladder tumour
VHR: very high risk

Bladder cancer is the sixth most common cancer diagnosis in males in the European Union, with >70% of cases attributed to non‐muscle‐invasive bladder cancer (NMIBC). Postoperative intravesical therapy remains a cornerstone of treatment, positioning NMIBC among the most financially burdensome malignancies [1]. To inform adjuvant treatment recommendations and surveillance schedules for NMIBC patients, since 2002 the European Association of Urology (EAU) has proposed patient stratification by prognostic risk groups. Accordingly, patients from the high‐risk groups are recommended BCG instillations (if not radical cystectomy [RC]). However, the risk stratification is underpinned by studies lacking BCG‐treated patients. In 2021, a Very High‐Risk group (VHR‐NMIBC) was added into the EAU guidelines as a result of the progression score model by Sylvester et al. [2]. We investigated the clinical outcomes of pre‐2021 High‐Risk NMIBC (HR‐NMIBC) patients treated with BCG and re‐classified them according to 2021 risk groups.

Patients with pre‐2021 HR‐NMIBC and treated with BCG were identified from the Bladder Cancer Prognosis Programme (BCPP, ethics approval 06/MRE04/65), a prospective cohort study recruiting newly‐diagnosed treatment‐naïve patients from 10 hospitals in the West Midlands (UK) from 2005 to 2011 [3]. Patient eligibility criteria included: primary Ta/T1 urothelial carcinomas of the urinary bladder, with or without concomitant carcinoma *in situ* (CIS); primary NMIBC treated with complete transurethral resection of bladder tumour (TURBT) and subsequent BCG (at least five instillations); minimum follow‐up of 3 months; no RC within 3 months from primary TURBT; high‐risk group (HR‐NMIBC) patients according to the 2020 EAU guidelines; patients with missing parameters only if the risk group would not change; and patients who completed at least one follow‐up cystoscopy after induction BCG instillations. Exclusion criteria: CIS without concomitant papillary tumour; previous history of muscle‐invasive disease; concomitant upper tract urothelial carcinoma; concomitant variant pathologies; immediate RC (<3 months) for NMIBC; patients treated with simultaneous chemotherapy instillation, systemic chemotherapy, radiation as well as other anti‐tumour treatments; less than five intravesical instillation doses of BCG; and disease recurrence before the 3‐month cystoscopy.

The primary outcome for the present study was progression‐free survival (PFS), defined as time from TURBT to the date of: increase in stage to ≥T2, metastasis, or death from bladder cancer. The secondary endpoint was recurrence‐free survival (RFS), recorded as time from TURBT to date of first bladder recurrence. Patients without a record of recurrence or progression were censored upon RC (as primary treatment for HR‐NMIBC) or the date of their last follow‐up visit. Kaplan–Meier curves for recurrence and progression were plotted to present survival status over time; the log‐rank statistic was used to assess differences in survival and predictive accuracy, quantified with Harrell's concordance index (C index).

A total of 212 BCG‐treated, pre‐2021 HR‐NMIBC cases were identified, of which 141 patients received adequate BCG (adequate BCG: induction plus two maintenance instillations, or two of six instillations in a second induction course; induction BCG: minimum five induction instillations [4]). The median (interquartile range [IQR]) follow‐up was 63.9 (46.4–74.3) months. Adequate‐BCG patients were younger than induction‐only patients, median (IQR) 70.2 (63.1–75.9) vs 73.3 (68.0–77.4) years (*P* = 0.004), whereas there were no differences in sex, ethnicity, post‐2021 EAU risk group, prostatic urethral involvement, single‐shot intravesical chemotherapy use, re‐TUR, or tumour grade, stage, size, multifocality, and CIS status.

For patients receiving induction‐only BCG, we found a statistically significant difference (*P* = 0.037) in PFS with worse outcomes for post‐2021 VHR‐NMIBC and a reasonable discriminatory ability (C index 0.66) with the tiered subgroups. The sub‐hazard ratio (sHR) was comparable between post‐2021 HR‐NMIBC and post‐2021 intermediate‐risk (IR)‐NMIBC (sHR 0.78, 95% CI 0.17–3.69) but worse for post‐2021 VHR‐NMIBC (sHR 2.66, 95% CI 0.58–12.2) (Fig. 1A). There was a trend toward poorer RFS for the post‐2021 VHR‐NMIBC group (sHR 2.17, 95% CI 0.62–7.58) compared to the post‐2021 IR‐NMIBC group (*P* = 0.068) (Fig. 1B).

**Fig. 1:**
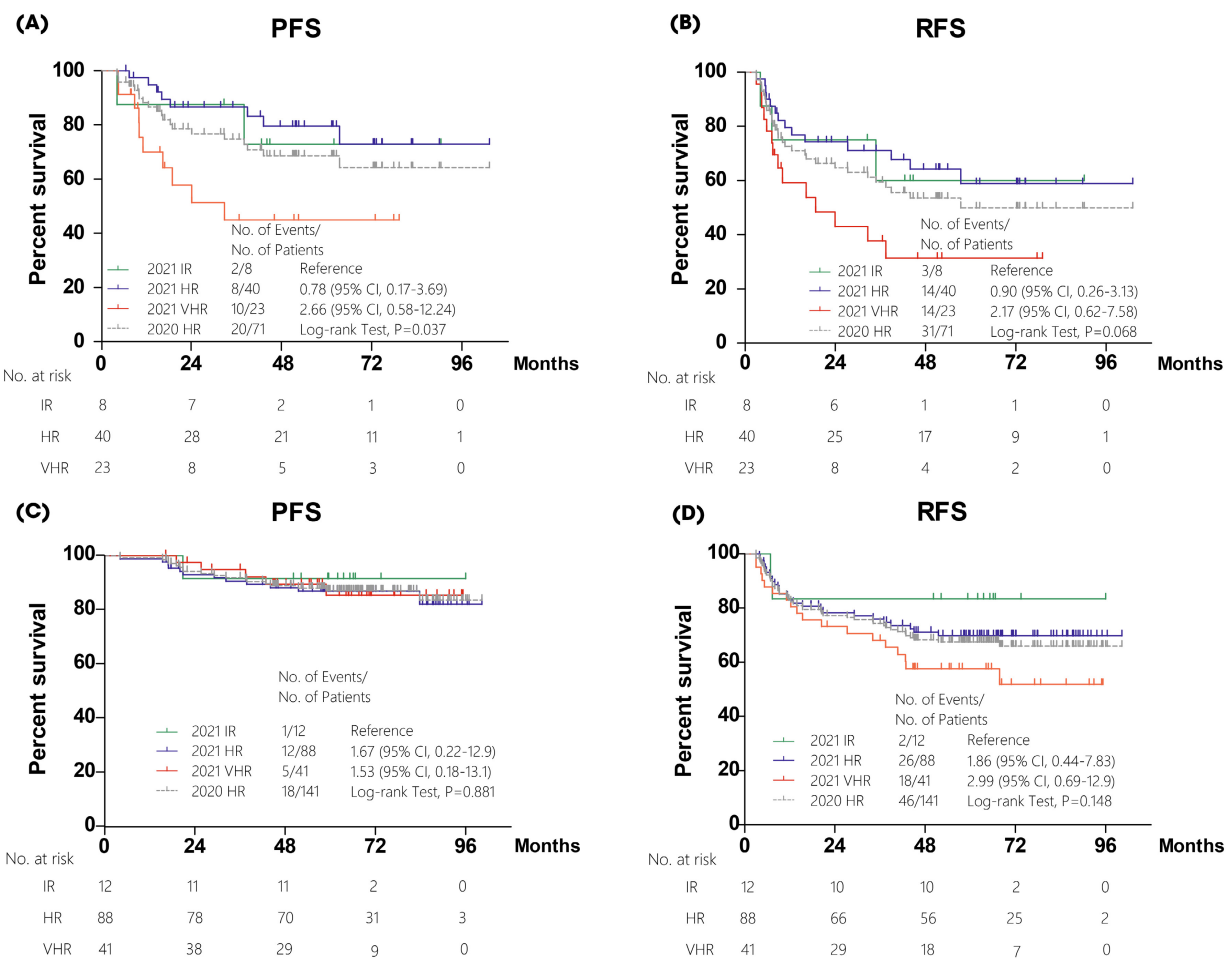
Kaplan–Meier analyses of time to progression and time to recurrence. Using IR patients as the reference, sHRs with 95% CIs were calculated for the HR and VHR groups based on the 2021 EAU classification. (A, B) Patients receiving induction‐only BCG therapy: (A) time to progression (sHR for HR‐NMIBC 0.78, 95% CI 0.17–3.69; for VHR‐NMIBC 2.66, 95% CI 0.58–12.2); (B) time to recurrence (sHR for HR‐NMIBC 0.90, 95% CI 0.26–3.13; for VHR‐NMIBC 2.17, 95% CI 0.62–7.58). (C, D) Patients receiving adequate BCG therapy: (C) time to progression (sHR for HR‐NMIBC 1.67, 95% CI 0.22–12.9; for VHR‐NMIBC 1.53, 95% CI 0.18–13.1); (D) time to recurrence (sHR for HR‐NMIBC 1.86, 95% CI 0.44–7.83; for VHR‐NMIBC 2.99, 95% CI 0.69–12.9). The cases represented by the 2020 HR group are shown with a dotted grey line in each plot for comparison.

In patients treated with adequate BCG, there were no significant differences in PFS for the post‐2021 HR‐NMIBC (sHR 1.67, 95% CI 0.22–12.9) and post‐2021 VHR‐NMIBC (sHR 1.53, 95% CI 0.18–13.1) groups compared to post‐2021 IR‐NMIBC (*P* = 0.881, C index 0.53). The Kaplan–Meier curves largely overlap and appeared no different to the pre‐2021 HR‐NMIBC curve (Fig. 1C). For RFS, there were no significant differences between the post‐2021 IR‐, HR‐, and VHR‐NMIBC groups (*P* = 0.148, C index 0.56), although the Kaplan–Meier curves all diverge from around 12 months (Fig. 1D).

Therefore, the post‐2021 EAU risk classification appears to be predictive for progression and recurrence for patients receiving induction‐only BCG. However, for patients receiving adequate BCG in our cohort, the post‐2021 IR‐, HR‐, and VHR‐NMIBC risk groups are not discriminatory for progression, although there may be borderline discrimination for recurrence events.

Our study is limited by a small cohort size. However, our data demonstrate a 5‐year progression probability for VHR‐NMIBC of 0.146 (95% CI 0.015–0.259) for patients receiving adequate BCG, consistent with that of Lobo et al. [5] (0.167, 95% CI 0.106–0.258); both studies demonstrate considerably lower risk than that reported by Sylvester et al. [2] (0.44, 95% CI 0.30–0.61) that excluded patients receiving BCG. The present study also validates the observation by Scilipoti et al. [6] that adequate BCG administration is linked to better PFS and RFS than patients not receiving adequate BCG.

Hence, in the context of adequate BCG therapy, we propose that upfront RC may represent overtreatment for patients with VHR‐NMIBC. Notwithstanding, more studies are needed to inform RC decisions, potentially incorporating molecular subtyping approaches [7] and circulating tumour DNA status [8].

## Disclosure of Interests

Richard T. Bryan is a paid consultant for Cystotech ApS (Denmark) and Nonacus Ltd (UK), and an unpaid charity trustee for Action Bladder Cancer UK (UK). He has contributed to advisory boards for AstraZeneca. Nicholas D. James has received honoraria from Sanofi, Bayer, Janssen, and Astellas Pharma with consulting or advisory roles for Sanofi, Bayer, Astellas Pharma, Janssen, Clovis Oncology, EUSA Pharma, and Pfizer. He has contributed to speakers’ bureau for Pierre Fabre, Ferring, Sanofi, Astellas Pharma, Janssen Oncology, Merck, and AstraZeneca and has received institutional research funding from Janssen, Astellas Pharma, Pfizer, Sanofi, Novartis, and AstraZeneca. The remaining authors have no disclosures.
